# Immunologic phenotype of patients with long-COVID syndrome of 1-year duration

**DOI:** 10.3389/fimmu.2022.920627

**Published:** 2022-08-24

**Authors:** Javier García-Abellán, Marta Fernández, Sergio Padilla, José Alberto García, Vanesa Agulló, Valle Lozano, Nuria Ena, Lidia García-Sánchez, Félix Gutiérrez, Mar Masiá

**Affiliations:** ^1^ Infectious Diseases Unit, Hospital General Universitario de Elche, Alicante, Spain; ^2^ Clinical Medicine Department, Universidad Miguel Hernández, Alicante, Spain; ^3^ CIBER de Enfermedades Infecciosas, Instituto de Salud Carlos III, Madrid, Spain; ^4^ Department of Clinical Chemistry, Hospital General Universitario de Elche, Alicante, Spain

**Keywords:** COVID-19, long COVID, post-COVID-19 syndrome, antibody response, antinuclear antibodies (ANA), one year, T-cell immune response, cellular immune response, humoral immune response

## Abstract

**Background:**

The pathophysiology of long-COVID remains unknown, and information is particularly limited for symptoms of very long duration. We aimed to assess the serological, T-cell immune responses and ANA titers of patients with long-COVID-19 syndrome of 1-year duration.

**Methods:**

Prospective, longitudinal study of hospitalized COVID-19 patients followed-up for 12 months. Sequential blood samples and COVID-19 symptom questionnaires (CSQ) were obtained, and humoral and cellular immune responses, antinuclear antibodies (ANA) and inflammation biomarkers were analyzed.

**Results:**

Of 154 patients discharged from hospital, 72 non-vaccinated with available CSQ in all visits were included. Of them, 14 (19.4%) reported persistent symptoms both at 6-months and 12-months, mainly asthenia (15.3%), myalgia (13.9%), and difficulty concentrating/memory loss (13.9%). Symptomatic patients were more frequently women, smokers, showed higher WHO severity score, and a trend to higher ICU admission. In the adjusted analysis, long-COVID syndrome was associated with lower frequency of detectable neutralizing antibodies (adjusted hazard ratio [aHR] 0.98; 95% confidence interval [CI], 0.97-0.99) and lower SARS-CoV-2-S1/S2 titers (aHR [95%CI] 0.14 [0.03–0.65]). T-cell immune response measured with a SARS-CoV-2-interferon-γ release assay was not different between groups. There was a higher frequency of positive ANA titers (≥160) in symptomatic patients (57.1% vs 29.3%, p=0.04), that was attenuated after adjustment aHR [95% CI] 3.37 [0.84-13.57], p=0.087. Levels of C-reactive protein and D-dimer were higher during follow-up in symptomatic patients, but with no differences at 12 months.

**Conclusion:**

Patients with 1-year duration long-COVID-19 syndrome exhibit a distinct immunologic phenotype that includes a poorer SARS-CoV-2 antibody response, low-degree chronic inflammation that tends to mitigate, and autoimmunity.

## Introduction

Following acute SARS-CoV-2 infection, a variable proportion of patients ranging from 10% to 80% report long-lasting symptoms involving one or multiple organs, a condition termed long-COVID, or post-COVID-19 syndrome when duration is longer than 12 weeks (1–3). An unexpectedly high percentage of patients remain symptomatic at 6 months after symptom onset (4, 5) and, although data are limited, even 12 months following acute infection (6, 7). The pathophysiology of this multisystem disease remains largely unknown. Dysregulated immune/inflammatory responses have been described several weeks after SARS-CoV-2 infection, some of which were found to occur more frequently in patients with long-COVID features (8–10). However, available information on the immune profile is scarce in patients with longer duration of the post-COVID-19 syndrome, and particularly in those who remain symptomatic 1 year after SARS-CoV-2 infection.

We aimed to characterize the immunologic phenotype, including humoral and cellular immune responses and the presence of autoantibodies, in patients with post-COVID-19 syndrome beyond 6 months and up to 1 year.

## Methods

We conducted a prospective, longitudinal study at Hospital General Universitario de Elche, Spain. All patients admitted for COVID-19 between March 10th and June 30th, 2020, with microbiologically confirmed infection through real-time polymerase chain reaction were initially included in the analysis. Patients were managed according to a predefined local protocol that included the diagnostic and therapeutic procedures during hospital stay and blood sampling for biochemical and sero-virological measurements at 1, 2, 6 and 12 months after discharge. Inflammatory biomarkers, including lymphocyte count, interleukin-6, ferritin, D-dimer, fibrinogen and C-reactive protein levels were measured at all visits. Lymphocyte count was measured by flow citometry (ADVIA ^®^ 2120i System, Siemens; normal range of 0.02 to 400 x 10^3 cell/µL); interleukin-6 was measured by electrochemiluminescence immunoassay (Cobas e411 System, Roche; normal range of 1.5 to 5000 pg/mL). Ferritin was analyzed using enhanced chemiluminescence immunoassay (VITROS^®^ 5600 System, Ortho Clinical Diagnostics; normal range of 1.25 to 1000 ng/mL). D-dimer was analyzed using particle-enhanced immunoturbidimetric assay (Sysmex CS-2500 System, Siemens; normal range of 0.17 to 4.40 mg/L). Fibrinogen was measured by clotting assay (Sysmex CS-2500 System,Siemens; normal range of 150 to 500 mg/dL). C-reactive protein was measured by immunoturbidimetric assay (VITROS^®^ 5600 System, Ortho Clinical Diagnostics; normal range of 0.24 to 330 mg/L).

Each visit, patients filled out a self-administered, self-rated COVID-19 symptom questionnaire (CSQ, Annex 1) comprising 11 items that patients graded using a 10-point increasing intensity scale (0=absence of the symptom and 10=the maximum perceived intensity of the symptom). We used a locally developed questionnaire because when we conducted the study no standardized validated questionnaire was available for the evaluation of long-COVID syndrome. Persistence of symptoms was defined as a score above the third quartile in any of the CSQ items both on 6-month and 12-month visits. Serum, EDTA plasma and whole blood specimens were obtained for measuring SARS-CoV-2–specific antibodies, neutralizing antibodies and interferon (IFN)-γ release assays, respectively.

IgG against the surface S1 domain of the spike protein (S-IgG) (Euroimmun, Lubeck, Germany) was measured at hospital admission and at 1, 2, 6 and 12 months after patients’ discharge, using commercial semi-quantitative EIA kits. Antibody levels were evaluated by calculating the ratio of the optical density (OD) of the patient sample over the OD of the calibrator (sample OD/calibrator OD= absorbance/cut-off [S/CO]). Results were interpreted according to the following criteria: ratio <1.1 was defined as negative and ratio ≥1.1 as positive. At the 12-month visit, S1- and S2-IgG antibody levels were measured using commercial immunoassay kits (LIAISON^®^ SARS-CoV-2 S1/S2 IgG assay, DiaSorin, Saluggia, Italy). Results were interpreted according to the following criteria: ratio <15 was defined as negative and ratio ≥15 as positive.

Detection of neutralizing antibodies against SARS-CoV-2 was performed at the 12-month visit in an automated instrument by means of a surrogate neutralizing antibody test (SARS-CoV-2 NeutraLISA, Euroimmun, Lübeck, Germany), that determines the inhibitory effect of antibodies that can compete with the biotinylated host-cell receptor (ACE2) for the binding to the receptor-binding domain (RBD) of the S1 subunit of SARS-CoV-2 spike protein (inhibition percentage, %IH). Results were interpreted as follows: %IH <35 was considered negative, and %IH ≥35 was considered positive.

SARS-CoV-2 cellular response was measured using a specific quantitative IFN-γ release assay in whole blood following the manufacture instructions (SARS-CoV-2 IGRA stimulation tube set, Euroimmun, Lübeck, Germany). Results were interpreted as follows: IFN-γ[SARS-CoV-2] - IFN-γ[blank] <100 mIU/mL was considered negative, 100-200 was considered borderline, and >200 was considered positive.

Detection of antinuclear antibodies was performed at the 12-month visit by indirect immunofluorescence assay (ANA-Mosaik 1A EUROPattern, Euroimmun, Lübeck, Germany) by automated incubation (IF Sprinter, Euroimmun) and assisted detection by EUROPattern Microscope (EUROLabOffice software). To increase the specificity of positive results, we considered positive ANA titers with a dilution ≥ 1/160.

Binomial logistic regression models were used to identify predictors of persistence of symptoms at 12 months. Covariates of interest with a p-value <0.05 in the crude comparison between groups and clinical relevant variables were included in multivariate analyses. To compare the curves of plasma biomarkers levels between groups, generalized additive mixed models were used. Interpolations in the graphs were carried out with cubic splines. Statistical analysis was performed using R-project version 3.6.2.

## Results

Of 166 hospitalized COVID-19 patients (148 non-critical and 18 admitted to the ICU), 154 were discharged and 123 (79.9%) had available follow-up with a filled questionnaire 1 year after admission, of whom 21 (17.1%) were excluded due to previous vaccination, and 30 (24.4%) because no data were available on the 6-month questionnaire leaving 72 patients for the final analysis. Of them, 14 (19.4%) reported persistent symptoms both at 6 and 12 months after admission, mainly asthenia (15.3%), myalgia (13.9%), difficulty concentrating and memory loss (13.9%), and insomnia (12.5%) (**Supplementary Table 1**). Characteristics of the patients according to the presence of 1-year long-COVID are shown in **Table 1**. Symptomatic patients were more frequently women (p=0.04), smokers (p=0.02), showed a higher score in the WHO ordinal severity scale on admission (p=0.02), and a trend to higher frequency of ICU admission (p=0.07).

**Table 1:** Clinical, serological and biomarker data according to the persistence of symptoms at 6 and 12 months after hospital admission for COVID-19.

Persistent symptoms at 6 and 12 months					
	Yes*	No	P value	Adjusted Hazard Ratio 95% CI	Adjusted P
Patients, no.	14 (19.4)	58 (80.6)			
Male sex	5 (35.7)	39 (67.2)	0.037	0.28 (0.11-0.70)	0.007
Age, years	59.5 (53-71)	60 (52-71)	0.938	–	–
Current smoking	3 (21.4)	1 (1.7)	0.021	12.02 (1.52-94.51)	0.01
Comorbidity, no. (%)				–	–
Any comorbidity#	10 (71.4)	38 (65.5)	0.761		
CCI, median (Q1, Q3) points	2 (1-3.5)	2 (1-3)	0.8		
Cardiovascular disease	3 (21.4)	10 (17.2)	0.708		
Hypertension	8 (57.1)	23 (39.7)	0.368		
Diabetes	3 (21.4)	8 (13.8)	0.438		
Chronic obstructive lung disease	0 (0)	2 (3.4)	1.000		
Autoimmune diseases	1 (7.1)	1 (1.7)	0.353		
Cancer	0 (0)	1 (1.7)	1.000		
WHO severity score			0.021	2.22 (1.41-3.50)	0.001
3 points	9 (64.3)	53 (91.4)			
4 points	1 (7.1)	0 (0)			
5 points	0 (0)	1 (1.7)			
6 points	4 (28.6)	4 (6.9)			
Bilateral lung infiltrates in CR	12 (85.7)	54 (93.1)	0.33	–	–
Length of hospital stay, days	11 (7-28.5)	11 (9-16)	0.852	–	–
Admission to the ICU	4 (28.6)	5 (8.6)	0.065	5.05 (1.62-15.76)	0.005
Immunomodulatory therapy^&^	9 (64.3)	40 (69)	0.756	–	–
Serological features					
SARS-CoV-2 S1/S2 IgG, AU/Ml	49 (16-96)	96.3 (46.4-133)	0.066	0.14 (0.03-0.65)	0.012
SARS-CoV-2 S-IgG (S/CO)	1.9 (1-4.3)	3.3 (1.9-4.6)	0.252	–	–
SARS-CoV-2-NeutraLISA, % IH	27.3 (16-75)	69.7 (39-83)	0.155	–	–
SARS-CoV-2-NeutraLISA positive, n (%)	6 (42.9)	46 (79.3)	0.007	0.98 (0.97-0.99)	0.023
SARS-CoV-2 IGRA, mIU/mL	1067 (341-1920)	1184 (544-2027)	0.739	–	–
SARS-CoV-2 IGRA positive, n (%)	11 (78.6)	49 (86)	0.524	–	–
Inflammatory biomarkers 12 months				–	–
Serum C-reactive protein, mg/L	1.8 (0.6-5.1)	1(0.6-3.4)	0.825		
Serum IL-6, pg/mL	3.6 (3.4-5.9)	3.4 (2.5-4.9)	0.466		
Serum Ferritin, ng/mL	49.3 (29-93.8)	66.2 (37.9-124)	0.453		
Serum D-dimer, mcg/mL	0.4 (0.2-0.8)	0.3 (0.2-0.4)	0.234		
Serum neutrophil/lymphocyte ratio	3.4 (3.1-4.6)	3.8 (3.1-4.5)	0.89		
Lymphocytes nadir count	0.6 (0.5-0.9)	0.9 (0.7-1.2)	0.065		
Antinuclear antibodies>1/160, n (%)	8 (57.1)	17 (29.3)	0.049	3.37 (0.84-13.57)	0.087

*Patients were allocated into the persistent symptomatic group if the score obtained in any of the symptoms of the Covid-19 Symptoms Questionnaire at the 6th and the 12th months was included in the top quartile. #This category included at least one of the following: diabetes, cardiovascular (including hypertension) respiratory, kidney, neurological disease, cirrhosis or malignant neoplasm. ^&^Immunomodulatory therapy included corticosteroids and/or tocilizumab. CCI, Charlson Comorbidity Index score; CR, Chest radiography; ICU, Intensive care unit; Q1, first quartile; Q3, third quartile; AU/mL, arbitrary units per mililiters, % IH, inhibition percentage, IGRA, Interferon-Gamma Release Assays; S/CO, absorbance/cut-off. IL-6, Interleukin-6. Summary statistics are provided as medians with interquartile ranges or numbers with percentages as appropriate.

**Figure 1:**
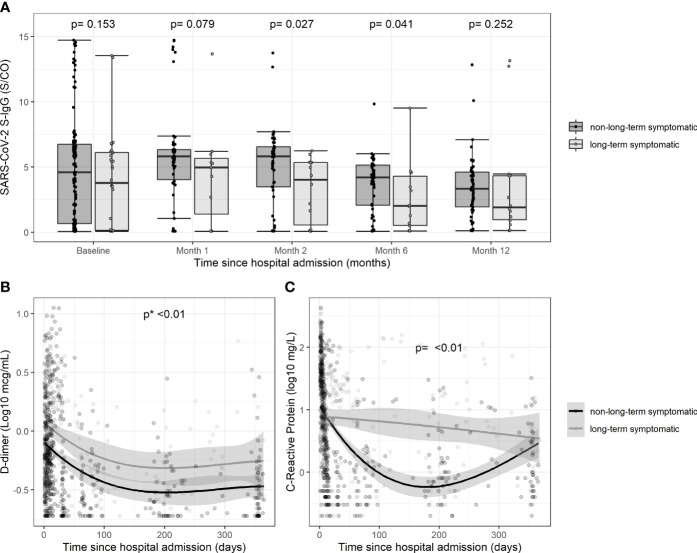
Temporal changes in the levels of antibodies and biomarkers during follow-up according to persistence of symptoms. **(A)**Serum titers of SARS-CoV-2 S-IgG on admission and at different time points after discharge according to the persistence of symptoms; **(B)** Serum levels of D-dimer during follow-up since hospital admission according to the persistence of symptoms; **(C)** Serum levels of C-reactive protein during follow-up since hospital admission according to the persistence of symptoms”.

The humoral immune responses are shown in **Table 1** and **Figure 1A**. The levels of S-IgG were lower in patients with post-COVID-19 syndrome during follow-up, with differences reaching statistical significance at months 2 and 6 after discharge compared to non-symptomatic patients (**Figure 1A**). At 12 months, the frequency of positive neutralizing antibodies was significantly lower in patients with post-COVID-19 syndrome, and titers of SARS-CoV-2 S1/S2 IgG tended to be lower. In the analysis adjusted for sex and ICU stay (both ICU stay and WHO severity score could not be simultaneously included in the model due to multicollinearity), the post-COVID-19 syndrome was associated with lower frequency of positive neutralizing antibodies, with adjusted hazard ratio (aHR) of 0.98 (95% confidence interval, CI, 0.97-0.99); and lower SARS-CoV-2 S1/S2 titers, with aHR (95% CI) of 0.14 (0.03–0.65). Sensitivity analyses replacing ICU stay for WHO ordinal scale on admission in the adjusted model showed similar results (data not shown).

T-cell immune response measured with a SARS-CoV-2 interferon-γ release assay was not different between groups with/without post-COVID-19 syndrome (**Table 1**).

There was a higher frequency of positive ANA titers (≥160) in patients with 1-year long-COVID features (57.1% vs 29.3%, p=0.04). Differences between groups remained, although were attenuated after adjustment (aHR [95% CI] 3.37 [0.84-13.57], p=0.087) (**Table 1**). A sensitivity analysis replacing ICU stay by previous autoinmmune diseases in adjustment showed similar results (aHR [95% CI] 1.01 [1-1.02], p=0.07). An additional sensitivity analysis excluding patients with prior autoimmune disease did neither differ substantially from the described results: 53.8% vs 28.1% positive ANA titers in participants with persistent vs those with non-persistent symptoms, respectively (p=0.07) and aHR (95% CI) of 3.31 (0.81-13.55), p=0.096. The most common pattern was nucleolar in 42.9% of patients.

**Figures 1B, C** shows the trajectories of the plasma concentrations of CRP and D-dimer. Levels of both biomarkers were higher during follow-up in patients with post-COVID-19 syndrome, although there were no differences at the 12-month visit. The trajectories of interleukin-6, ferritin and the neutrophil-to-lymphocyte ratio were not different between groups (data not shown).

## Discussion

Our findings suggest a dysregulated immune response in hospitalized patients with long-COVID-19 syndrome of 1-year duration, consisting of decreased frequency of detectable neutralizing antibodies, decreased anti-spike antibody levels, and higher frequency of positive ANA titers. No abnormal findings were however observed in T-cell responses measured with a SARS-CoV-2 interferon-γ release assay. Convalescent patients also showed a differential trajectory of inflammation biomarkers, consisting of higher levels of C-reactive protein (CRP) and D-dimer, although with no differences at month 12.

The poorer antibody response observed in patients with 1-year long-COVID-19 syndrome is in line with our previous findings in convalescents with ongoing symptoms 6 months after hospital admission, although neutralizing antibodies, T-cell immunity and ANA titers were not measured in that study (11). The mechanisms involved in the inferior humoral response are unknown. Severe acute SARS-CoV-2 infection has been associated with impaired formation of germinal centers and a striking reduction in Bcl-6-expressing B cells, leading to dysregulated SARS-CoV-2-specific humoral immunity and systemic inflammation (12). Patients developing post-COVID-19 syndrome in our study were more severely ill, and might potentially have had greater and/or long-lasting damaging effect on germinal centers, leading to defective recovery of protective immunity against SARS-CoV-2. Long-COVID has been associated with decreased concentrations of the IgG3 subclass immunoglobulin, both during acute infection and at 6 months (13). The IgG3, along with IgG1, constitute the predominant antibody responses against several viral diseases, including SARS-CoV-2 (14). Patients with persistent symptoms 3 months after infection were described as having neutrophil dysfunction that tended to interfere with the production of anti-SARS-CoV-2-S1 neutralizing antibodies (15). The presence of anti-idiotype antibodies against SARS-CoV-2 S-IgG has recently been proposed as a mechanism of down-regulation of the specific humoral response by binding to protective neutralizing antibodies, resulting in immune-complex formation and clearance. These anti-idiotype antibodies have also been related with the persistence of symptoms in long-COVID and possible vaccines´ adverse effects (16).

An impaired humoral immune response, particularly when involving the protective neutralizing antibodies, might favor persistent SARS-CoV-2 infection or antigenic reservoir (17), as well as immune stimulation, sustained inflammation and auto-reactivity (18), which may contribute to perpetuation of symptoms. Accordingly, a deficient humoral response might be linked with the increased frequency of positive ANA levels in our long-COVID patients. SARS-CoV-2 may act as a triggering factor for the development of a rapid autoimmune autoinflammatory dysregulation. Positive ANA have been detected in up to 50% of patients with acute SARS-CoV-2 infection (19, 20) but, conversely, long-term data are limited and contradictory (21, 22). ANAs have been suggested to play a pathogenic role in disease through different mechanisms, including the formation and deposition of immune complexes containing ANA and nuclear autoantigen, molecular mimicry and direct interaction on target cells or penetration into cells (23). It remains to be determined whether they might potentially contribute to the severity and persistence of symptoms associated with COVID-19. Our results are in agreement with findings from Seeßle et al, who described a higher proportion of positive ANA titers 12 months after COVID-19, particularly in association with neurocognitive symptoms (21).

We did not find differences in T-cell responses measured with an interferon-γ release assay according to the occurrence of 1-year post-COVID-19 syndrome. Although not measured in our study, the levels of cytotoxic CD8+ T cells have been found to be increased and activated in patients with long-COVID up to 8 months after acute infection (24, 25), and activation of CD8+ T cells has been associated with autoimmunity (26, 27) and with enhanced ability to produce inflammatory mediators (28). Our results suggest ongoing chronic inflammation lasting up to one year in patients with long-COVID, a longer period than that described so far (24, 29).

The sample size is a limitation of the study. In the absence of a definition of long-COVID, we selected patients with the highest CSQ scores, and therefore those with milder symptoms are not represented. Neutralizing antibodies could not be determined at the beginning of the study to analyze neutralization kinetics between baseline and 12 month visit, since the SARS-CoV-2 NeutraLISA test was not initially available. Our study, however, provides long-term longitudinal data of a prospective cohort of long-COVID patients with close and thorough follow-up, and comprehensive characterization of the 12-month immune responses.

In conclusion, patients with 1-year duration long-COVID-19 syndrome exhibit a distinct immunologic phenotype that includes decreased levels of anti-SARS-CoV-2 anti-spike and neutralizing antibodies, autoimmunity, and low-degree chronic inflammation that tends to dissipate. Although these findings have yet to be confirmed in larger cohorts, they may contribute to deepen into the pathogenesis of long-COVID.

## Data availability statement

The original contributions presented in the study are included in the article/**Supplementary Material**. Further inquiries can be directed to the corresponding authors.

## Ethics statement

The studies involving human participants were reviewed and approved by Ethics Committee of Hospital General Universitario de Elche. The patients/participants provided their written informed consent to participate in this study.

## Author contributions

JG-A: investigation, writing—original draft preparation & editing. MF, VA, VL: Investigation, Writing – review & editing SP: software, and formal analysis, writing – review & editing. JG: formal analysis and writing—review and editing. NE, LG-S: data curation and writing—review and editing. Investigation and writing—review and editing. FG and MM: conceptualization, methodology, writing—original draft preparation, reviewing and editing, and supervision. All authors contributed to the article and approved the submitted version.

## Funding

This work was supported by the RD16/0025/0038 and CB21/13/00011 projects of the Plan Nacional Research+Development+Innovation (R+D+I) and cofinanced by Instituto de Salud Carlos III - Subdirección General de Evaluación y Fondo Europeo de Desarrollo Regional (grants PI16/01740, PI18/01861, CM19/00160, CM20/00066, COV20/00005), ILISABIO (A-32 2020) and AICO/2021/205.

## Conflict of interest

The authors declare that the research was conducted in the absence of any commercial or financial relationships that could be construed as a potential conflict of interest.

## Publisher’s note

All claims expressed in this article are solely those of the authors and do not necessarily represent those of their affiliated organizations, or those of the publisher, the editors and the reviewers. Any product that may be evaluated in this article, or claim that may be made by its manufacturer, is not guaranteed or endorsed by the publisher.
