# Supplementary material for: Immunologic phenotype of patients with long-COVID syndrome of 1-year duration
Source: Front Immunol. 2022 Aug 24;13:920627. doi: 10.3389/fimmu.2022.920627 (PMC9451924; doi:10.3389/fimmu.2022.920627)

**Supplemental Table 1. Clinical characteristics of patients with persistent COVID one year after discharge.**

| **Variable** |  |  | |
| --- | --- | --- | --- |
| **Patients with available questionnaire at 6 and 12 months, n.**    Symptomatic at 6 and 12 months visits  No persistence of symptoms |  | 72  14  58 | (100)  (19.4)  (80.6) |
| **Persistent symptoms at 12 months**    Asthenia/fatigue  Myalgias/arthralgias  Memory loss / trouble concentrating  Insomnia  Headache  Dyspnoea  Digestive symptoms (nausea/vomiting/diarrhea)  Depression/anxiety  Heart palpitations  Ageusia/anosmia  Nasal congestion/expectoration  Sore throat  Hair loss (alopecia)  Cough  Sweating |  | 11  10  10  9  8  6  6  6  6  5  4  4  4  3  3 | (15.3)  (13.9)  (13.9)  (12.5)  (11.1)  (8.3)  (8.3)  (8.3)  (8.3)  (6.9)  (5.6)  (5.6)  (5.6)  (4.2)  (4.2) |
| **Number of symptoms per patient**  1 symptom  2 symptoms  3 symptoms  4 symptoms  5 or more symptoms |  | 1  2  2  3  6 | (1.4)  (2.8)  (2.8)  (4.2)  (8.3) |

**Annex1. Self-reported COVID-19 symptoms questionnaire**


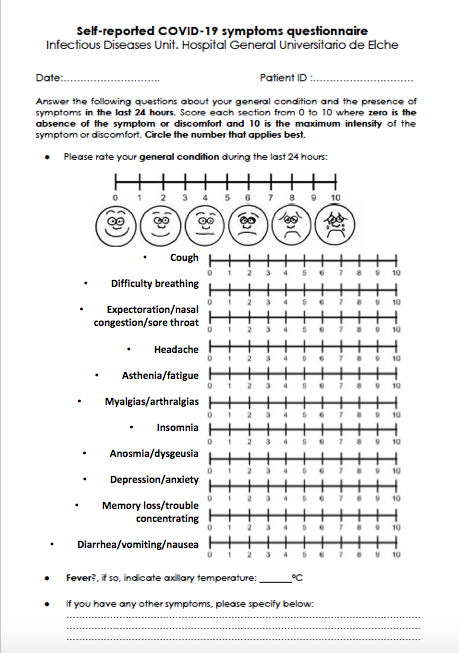

Supplement: Supplementary file 1 [file Table_1.docx]
